# Radiation dose reduction for CT assessment of urolithiasis using iterative reconstruction: A prospective intra-individual study

**DOI:** 10.1007/s00330-017-4929-2

**Published:** 2017-07-10

**Authors:** Annemarie M. den Harder, Martin J. Willemink, Pieter J. van Doormaal, Frank J. Wessels, M. T. W. T. Lock, Arnold M. R. Schilham, Ricardo P. J. Budde, Tim Leiner, Pim A. de Jong

**Affiliations:** 1 Department of Radiology, Utrecht University Medical Center, P.O. Box 85500, E01.132, 3508GA Utrecht, The Netherlands; 2 Department of Radiology, Erasmus Medical Center, P.O. Box 2040, 3000CA Rotterdam, The Netherlands; 3 Department of Urology, University Medical Center, P.O. Box 85500, 3508GA Utrecht, The Netherlands

**Keywords:** Urolithiasis, Multidetector computed tomography, Radiation ionising, Sensitivity, Imaging, Diagnostic

## Abstract

**Objective:**

To assess the performance of hybrid (HIR) and model-based iterative reconstruction (MIR) in patients with urolithiasis at reduced-dose computed tomography (CT).

**Methods:**

Twenty patients scheduled for unenhanced abdominal CT for follow-up of urolithiasis were prospectively included. Routine dose acquisition was followed by three low-dose acquisitions at 40%, 60% and 80% reduced doses. All images were reconstructed with filtered back projection (FBP), HIR and MIR. Urolithiasis detection rates, gall bladder, appendix and rectosigmoid evaluation and overall subjective image quality were evaluated by two observers.

**Results:**

74 stones were present in 17 patients. Half the stones were not detected on FBP at the lowest dose level, but this improved with MIR to a sensitivity of 100%. HIR resulted in a slight decrease in sensitivity at the lowest dose to 72%, but outperformed FBP. Evaluation of other structures with HIR at 40% and with MIR at 60% dose reductions was comparable to FBP at routine dose, but 80% dose reduction resulted in non-evaluable images.

**Conclusions:**

CT radiation dose for urolithiasis detection can be safely reduced by 40 (HIR)–60 (MIR) % without affecting assessment of urolithiasis, possible extra-urinary tract pathology or overall image quality.

**Key Points:**

*• Iterative reconstruction can be used to substantially lower the radiation dose.*

*• This allows for radiation reduction without affecting sensitivity of stone detection.*

*• Possible extra-urinary tract pathology evaluation is feasible at 40–60% reduced dose.*

**Electronic supplementary material:**

The online version of this article (doi:10.1007/s00330-017-4929-2) contains supplementary material, which is available to authorized users.

## Introduction

The most frequent cause of acute flank pain is urolithiasis, which affects 3–5% of the population [1]. The risk of recurrence of urolithiasis is high, with 50% after 5 years and more than 72% after 20 years [2]. In the USA, the total estimated annual cost for stone disease was over US$10.3 billion in 2006, an almost fivefold increase in 6 years [3]. This rise is still present today. Unenhanced computed tomography (CT) is often used for the diagnosis of urolithiasis. Compared to ultrasonography, CT offers the possibility to detect extra-urinary causes of flank pain [1], and has a higher specificity and sensitivity. The guidelines of the European Association of Urology [4] as well as the American College of Radiology [5] advise using low-dose CT in patients with acute disease and suspicion of urolithiasis, while the American Urological Association provides no clear recommendation [6]. However, CT suffers from several disadvantages such as higher costs compared to ultrasonography, limited availability in developing countries and the associated exposure to ionising radiation. Technical advancements like iterative reconstruction (IR) algorithms have resulted in substantial radiation dose reductions. IR results in reduced noise, allowing acquisition of images at reduced radiation dose levels without intrinsically hampering image quality. Several studies have investigated the potential of IR for unenhanced abdominal CT, with most studies focusing on urinary stone detection and image quality [7–10]. The purpose of this study was to assess the performance of hybrid IR (HIR) and model-based IR (MIR) in patients with urolithiasis at reduced-dose CT.

## Materials and methods

This prospective study was approved by our local institutional review board (NL46146.041.13). Inclusion criteria were: patient age of ≥40 years and a scheduled unenhanced abdominal CT for follow-up of urolithiasis. Patients with concomitant participation in another study with x-ray exposure were excluded. All patients were informed about the additional radiation exposure and provided written informed consent. Gender, age, height and weight of all patients were recorded. Twenty patients were included between April 2014 and October 2015.

### CT acquisition

All acquisitions were performed on a 256-slice CT system (Brilliance iCT, Philips Healthcare, Best, The Netherlands). The routine dose acquisition was followed by three low-dose acquisitions with the same scan length during the same session. A tube voltage of 120 kVp was used for all acquisitions. To create different dose levels, the tube current was lowered. All patients were imaged with automatic exposure control using a reference of 100 mAs (routine dose) and 60, 40 and 20 mAs (reduced dose levels), respectively. The pitch was 0.915 with a rotation time of 0.4 s. Slices were reconstructed with a thickness of 0.9 mm and increment of 0.7 mm for all measurements. All acquisitions were reconstructed with filtered back projection (FBP), HIR (iDose^4^ level 4, Philips Healthcare) and MIR (IMR level 2, Philips Healthcare). The routinely used kernel B was used for FBP and iDose^4^ reconstructions. IMR has three groups of kernels, namely Body Soft Tissue, Body Routine and Body SharpPlus [11]. Both Body Soft Tissue and Body Routine are recommended by the vendor for soft tissue evaluation, and were therefore both used. Dose length product (DLP) and volumetric CT dose index (CTDI_vol_) were recorded for each scan. The effective dose was calculated by multiplying the DLP with the conversion factor for 120 kV abdominal acquisitions (0.0153 mSv/(mGy×cm)) [12].

### Image evaluation

All FBP acquisitions at routine dose were assessed to set the reference for the number and location of the stones. Urolithiasis was classified as stones (kidney, ureter or bladder), papillary calcifications or parenchymal calcifications. The reference evaluation was done by a certified board radiologist (PJ) with over 10 years of experience in abdominal radiology. This radiologist was not an observer in the current study. The maximal size of all stones was measured in three planes (transversal, coronal and sagittal) using the routine dose acquisition reconstructed with FBP and the average maximal diameter was calculated [13]. Subsequently, two observers (FW and PD) with more than 5 years of experience in abdominal radiology assessed the number of stones on all acquisitions and reconstructions.

Subjective image quality was assessed by the same two observers who were blinded for patient characteristics, acquisition and reconstruction information, and images were evaluated in randomised order. Overall image quality was scored using a 4-point Likert scale:

- Score 1: poor image quality – not diagnostically acceptable for interpretation;
- Score 2: suboptimal image quality – partially non-diagnostic;
- Score 3: acceptable image quality – diagnostic interpretation possible;
- Score 4: excellent image quality.

A score of 1–2 was considered unacceptable. One patient was used as a test case and used for training in a consensus meeting. Because it is important to be able to diagnose extra-urinary tract pathology, several other aspects were scored as well. Observers were asked to assess if there was a prior cholecystectomy, or, if the gall bladder was still present, it was assessed for the presence stones or wall thickening, or whether these could have been visualized if present. The maximal infra-renal diameter of the aorta was assessed in the anterior-posterior direction for abdominal aneurysm evaluation. The sigmoid and appendix were evaluated to see if there were signs of diverticulitis or appendicitis, or whether these could have been visualized if present. The maximal width of the body of the adrenal gland was assessed on the transversal plane, and the average Hounsfield unit (HU) was derived by drawing a circular region of interest (ROI) as large as possible.

Objective image quality was measured by drawing a circular ROI in the renal cortex (both left and right), aorta, retroperitoneal fat and air in the bowel. Noise was defined as the standard deviation (SD) of the HU measurement in the ROI.

### Statistical analysis

Statistical analyses were performed using SPSS (version 20.0, IBM, New York, USA). The test case was excluded from further analysis. FBP at routine dose was used as a reference standard, and all data were compared to the reference standard. To assess the subjective overall image quality the scores of the two observers were averaged. Also, the number of unacceptable scans (score 1 or 2) was calculated per observer. Sensitivity for the detection of stones was calculated by dividing the number of correctly identified stones by the total number of stones and multiplying this number by 100%. The sensitivity as well as the number of false positives was calculated both on a patient level and overall. In the per-patient level analysis, the number of false positives and the sensitivity were calculated for each patient separately. Therefore, missing a stone in a patient with only 1 stone will have a heavier weight than missing a stone in a patient with multiple stones. In the overall analysis the total number of false positives for all patients combined was calculated per observer, as well as the overall sensitivity. In this analysis, each stone has an equal weight.

Data were compared using the Friedman test and post-hoc analyses were performed using the Wilcoxon signed rank test. A *p*-value below 0.05 was considered significant. For the post-hoc tests a Bonferroni corrected *p*-value of 0.0125 was used. Values are presented as medians (interquartile range, IQR) unless stated otherwise.

Interobserver agreement was measured using Cohen’s kappa coefficient and the percentage of agreement for categorical variables. The kappa was interpreted as poor (0.00 ≤ k ≤ 0.20), fair (0.21 ≤ k ≤ 0.40), moderate (0.41 ≤ k ≤ 0.60), good (0.61 ≤ k ≤ 0.80) or excellent (0.81 ≤ k ≤ 1.00). For categorical variables with more than two categories a weighted kappa was used. A two-way random intraclass correlation coefficient (ICC) was used for continuous parameters, as well as the difference (in mm or HU).

## Results

A total of 20 patients were included, of which one patient was used as a test case for the consensus meeting. Therefore, ultimately 19 patients were investigated: 16 males (16/19, 84%) and three females (3/19, 16%). The median age was 61 (IQR 52–67) years. Mean height was 181 (172–189) cm with a weight of 86 (75–100) kg resulting in a body mass index (BMI) of 25.6 (25.0–32.4) kg/m^2^.

Effective dose was 4.8 (4.1–7.8) mSv at routine dose and 2.8 (2.5–4.7), 1.9 (1.6–3.1) and 0.9 (0.8–1.5) mSv at reduced dose levels, respectively. CTDI_vol_ was 6.6 (5.5–10.5), 3.9 (3.3–6.3), 2.6 (2.2–4.2) and 1.3 (1.1–2.1) mGy, respectively, while the DLP was 312 (270–508) mGy*cm at routine dose and 184 (162–304), 123 (106–204) and 60 (51–99) mGy*cm at reduced dose levels, respectively.

### Diagnostic performance for stones and calcifications

In total 74 stones were present in 17 patients, including 63 stones, seven papillary calcifications and four parenchymal calcifications (Table 1). Of the 63 stones, 62 were in the kidney and one in the ureter. The size of the stones was smaller than 3 mm (19/74, 26%), 3–5 mm (26/74, 35%), 5–10 mm (21/74, 28%) or larger than 10 mm (8/74, 11%).

**Table 1:** Stone and calcification characteristics

	Diameter								Total
	<3 mm		3–5 mm		5–10 mm		>10 mm		
	n	Density (HU)	n	Density (HU)	n	Density (HU)	n	Density (HU)	
Stone	16	307 (251–371)	23	365 (323–531)	16	720 (541–897)	8	822 (665–982)	63
Papillary calcification	2	333 (257–333)	1	335	4	837 (645–1237)	0	NA	7
Parenchymal calcification	1	481	2	312 (258–312)	1	694	0	NA	4
Total	19		26		21		8		74

**Fig. 1:**
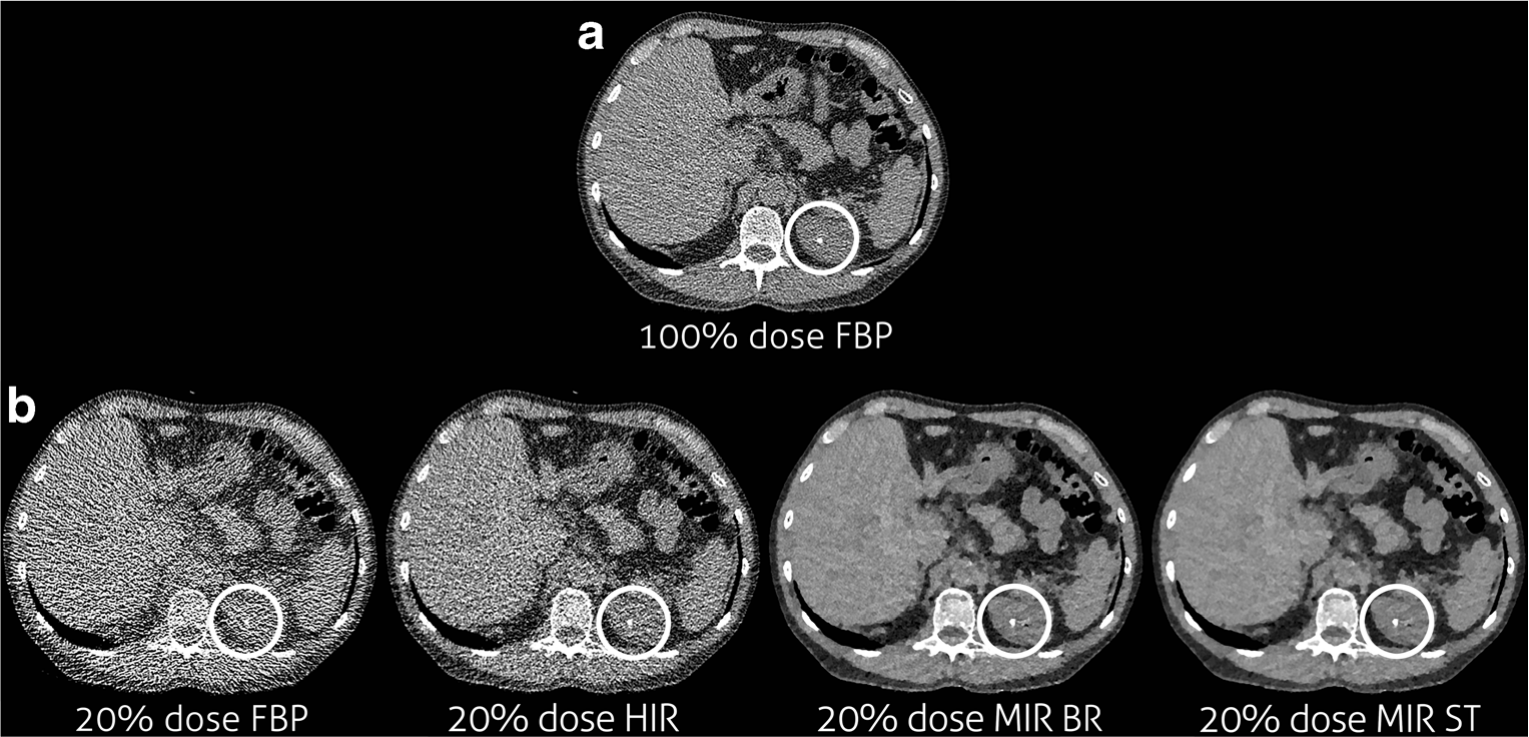
Example of decreased sensitivity for stone detection. From left to right the stone at routine dose reconstructed with FBP (**a**), the FBP reconstruction at the lowest dose level on which the stone was missed (**b**) and the IR reconstructions (HIR, MIR Body Routine and MIR Soft Tissue) at the same dose level on which the stone is clearly visible

The accuracy of stone detection is shown in Table 2. The sensitivity at routine dose was 94% with FBP, because some stones were missed by a single observer. The sensitivity at routine dose with IR was 100%. At reduced dose, the sensitivity with FBP decreased to 89%, 88% and 50%, respectively. While with HIR the sensitivity was 100%, 89% and 72%, respectively. MIR Body Routine resulted in a sensitivity of 100% at all dose levels while the sensitivity with MIR Soft Tissue reduced to 79% at the lowest dose level. An example of decreased sensitivity is provided in Fig. 1. All missed stones concerned stones with a size below 3 mm. The number of false positives was low at all dose levels with a median number between 0 and 1. The overall sensitivity is presented in Table A in the Appendix.

**Table 2:** Diagnostic performance. The sensitivity for stone detection was calculated on a patient level, and stones, papillary calcifications and parenchymal calcifications were combined. Two patients did not have any stones. The sensitivity is presented as median (interquartile). For the assessment of extra-urinary tract pathology the number [percentage] of non-assessable reconstructions is displayed per observer

	Sensitivity	Cholecys-tectomy		Gall bladder stones		Gall bladder wall thickening		Sigmoid diverticulitis		Appendix visible		Appendicitis	
Routine dose		Obs. 1	Obs. 2	Obs. 1	Obs. 2	Obs. 1	Obs. 2	Obs. 1	Obs. 2	Obs. 1	Obs. 2	Obs. 1	Obs. 2
FBP	94.4 (80.0–100.0)	0 [0]	0 [0]	1 [5]	1 [5]	1 [5]	8 [42]	0 [0]	0 [0]	0 [0]	1 [5]	0 [0]	2 [11]
HIR	100.0 (80.0–100.0)	0 [0]	0 [0]	1 [5]	1 [5]	2 [11]	2 [11]	0 [0]	0 [0]	0 [0]	1 [5]	0 [0]	1 [5]
MIR (BR)	100.0 (92.9–100.0)	0 [0]	0 [0]	0 [0]	1 [5]	0 [0]	3 [16]	0 [0]	0 [0]	1 [5]	1 [5]	1 [5]	1 [5]
MIR (ST)	100.0 (77.8–100.0)	0 [0]	0 [0]	1 [5]	1 [5]	1 [5]	2 [11]	0 [0]	0 [0]	2 [11]	1 [5]	1 [5]	2 [11]
40% reduced dose													
FBP	88.9 (50.0–100.0)	0 [0]	0 [0]	1 [5]	2 [11]	4 [21]	16 [84]	0 [0]	0 [0]	0 [0]	2 [11]	0 [0]	3 [16]
HIR	100.0 (80.0–100.0)	0 [0]	0 [0]	1 [5]	1 [5]	4 [21]	8 [42]	0 [0]	0 [0]	1 [5]	1 [5]	1 [5]	2 [11]
MIR (BR)	100.0 (92.9–100.0)	0 [0]	0 [0]	1 [5]	1 [5]	1 [5]	4 [21]	0 [0]	0 [0]	0 [0]	1 [5]	0 [0]	2 [11]
MIR (ST)	100.0 (92.9–100.0)	0 [0]	0 [0]	1 [5]	1 [5]	1 [5]	5 [26]	0 [0]	0 [0]	1 [5]	0 [0]	2 [5]	2 [11]
60% reduced dose													
FBP	87.5 (50.0–100.0)	0 [0]	0 [0]	2 [11]	5 [26]	7 [37]	19 [100]	0 [0]	0 [0]	2 [11]	4 [21]	4 [21]	5 [26]
HIR	88.9 (75.0–100.0)	0 [0]	0 [0]	1 [5]	2 [11]	5 [26]	13 [68]	0 [0]	0 [0]	1 [5]	1 [5]	0 [0]	5 [26]
MIR (BR)	100.0 (81.3–100.0)	0 [0]	0 [0]	1 [5]	1 [5]	2 [11]	8 [42]	0 [0]	0 [0]	0 [0]	1 [5]	0 [0]	1 [5]
MIR (ST)	100.0 (80.0–100.0)	0 [0]	0 [0]	1 [5]	1 [5]	3 [16]	4 [21]	1 [5]	0 [0]	1 [5]	1 [5]	1 [5]	1 [5]
80% reduced dose													
FBP	50.0 (16.7–100.0)	1 [5]	3 [15]	14 [74]	14 [74]	18 [95]	10 [100]	17 [90]	8 [42]	16 [84]	16 [84]	18 [95]	16 [84]
HIR	72.2 (62.5–100.0)	0 [0]	0 [0]	4 [21]	6 [32]	11 [58]	18 [95]	1 [5]	0 [0]	6 [32]	3 [16]	4 [21]	6 [32]
MIR (BR)	100.0 (68.8–100.0)	0 [0]	0 [0]	2 [11]	3 [16]	4 [21]	9 [47]	1 [5]	0 [0]	1 [5]	1 [5]	1 [5]	4 [21]
MIR (ST)	78.6 (50.0–100.0)	0 [0]	0 [0]	1 [5]	1 [5]	4 [21]	11 [58]	0 [0]	0 [0]	0 [0]	2 [11]	2 [11]	2 [11]

*FBP f*iltered back projection*, HIR* hybrid iterative reconstruction*, MIR* model-based iterative reconstruction*, BR* body routine, *ST* soft tissue, *Obs* observer

### Extra-urinary tract pathology

Results of the assessment of extra-urinary tract pathology are displayed in Table 2. At routine dose reconstructed with FBP, the assessment of prior cholecystectomy and sigmoid diverticulitis was possible in all patients. Gall bladder assessment for stones and for wall thickening was hampered in one patient (5%) and one or eight patients (5% or 42%), respectively, depending on the observer. One observer also scored the appendix as not assessable (one patient, 5%) or as inflammatory signs not assessable (two patients, 11%). The assessment of prior cholecystectomy and sigmoid diverticulitis was possible up to 60% reduced dose with FBP, while with HIR and MIR the dose could be decreased by 80%. Gall bladder stones were still assessable at 60% reduced dose with both MIR kernels, and at 80% dose using MIR Soft Tissue. The assessment of gall bladder wall thickening was similar to the reference at 40% reduced dose using MIR (both kernels). While for the assessment of the appendix the dose could be reduced by 40% for HIR and by 60% using MIR (both kernels). Overall, 40% dose reduction with HIR and 60% dose reduction with MIR yielded similar results to the reference (routine dose with FBP).

The aorta and adrenal gland measurements did not show any significant differences at reduced dose (Appendix, Table B). However, with FBP the adrenal glands were not always assessable at all reduced dose levels, while at 60% dose reduced-dose HIR resulted in not assessable reconstructions as well. At the lowest dose level, the adrenal gland was not assessable in several patients on all reconstruction methods (Appendix, Table B).

Observer agreement for subjective parameters was moderate to excellent, except for gallbladder wall thickening with a kappa of 0.31. The ICC was excellent for measuring the diameter of the aorta (ICC 0.95), and poor to fair for the adrenal gland measurements. Differences between observers were low with a mean difference of 1.2 and 0.2 mm and 3.9 and 7.5 HU for the left and right adrenal gland respectively. Results are also shown in the Appendix, Table C.

### Image quality

**Fig. 2:**
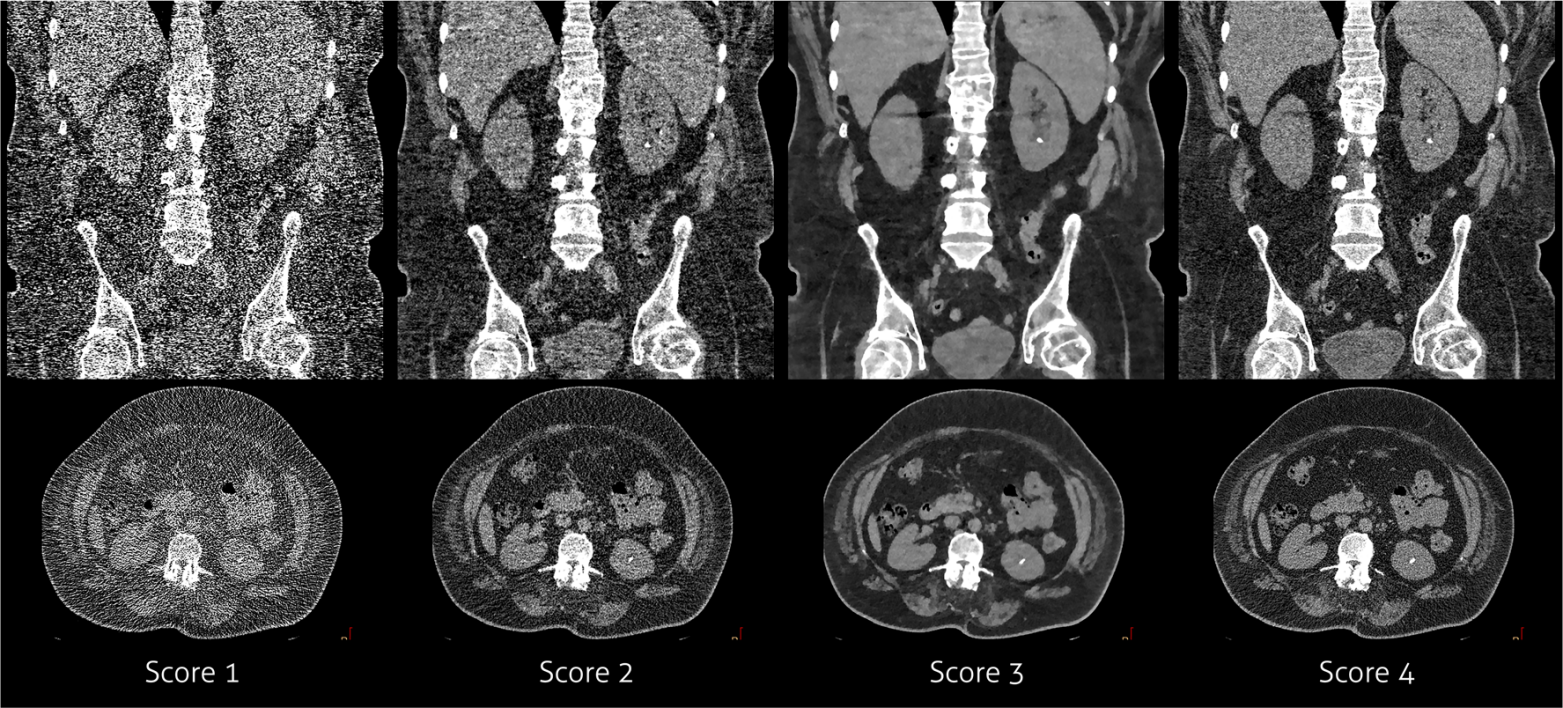
Example of the subjective image quality score. From left to right the different scores: score 1 with FBP at 80% reduced dose, score 2 with HIR at 80% reduced dose, score 3 with MIR Soft Tissue at 60% reduced dose and score 4 with HIR at the routine dose level. Note that the kidney stone can be seen in all images. Score 1 was mainly because of excessive noise. Score 2 was also due to substantial noise. Score 3 was given because of smoothening by IR. Score 4 contains some noise, but radiologists are used to some noise and tend to prefer this to extensive smoothening and noise reduction

An example of the different subjective image quality scores is provided in Fig. 2. An additional example can be found in the Appendix, Figure A *.* The image quality scores are provided in Table 3. The median image quality score at routine dose reconstructed with FBP was 3 (acceptable image quality). One observer scored all scans at routine dose as acceptable image quality, while the other observer scored nine patients (47%) as having unacceptable image quality. HIR, MIR Body Routine and MIR Soft Tissue resulted in significantly higher subjective image quality scores of 4 on routine dose. In addition, the number of patients with unacceptable image quality decreased to one (5%) with HIR and to zero with both MIR kernels. With FBP the image quality significantly decreased at the two lowest dose levels. At the lowest dose level, the image quality was unacceptable in all patients with FBP. At 40% and 60% reduced dose, IR resulted in similar or improved image quality compared to the reference acquisition (FBP at routine dose). While at the lowest dose level, the image quality significantly decreased with all reconstruction techniques; however, the image quality was still better with HIR (2) and MIR (3) compared to FBP (1).

**Table 3:** Subjective image quality and noise per reconstruction method and per dose level. For the image quality, the average score of the two observers was used. Those data are presented as median [interquartiles]. Also, the number of examinations [%] with unacceptable image quality (score 1 or 2) is shown per observer. The noise is presented as medians (interquartiles)

	Subjective image quality	Unacceptable image quality		Renal cortex (right)	Renal cortex (left)	Aorta	Retroperitoneal fat	Air
Routine dose		Obs. 1	Obs. 2					
FBP	3 [3–3]	0 [0]	9 [47]	53.3 (48.6–61.2) *Reference*	51.8 (42.8–60.0) *Reference*	56.5 (50.3–68.8) *Reference*	54.7 (45.7–61.4) *Reference*	23.5 (22.4–26.3) *Reference*
iDose^4^	4 [4–4]*	0 [0]	1 [5]	32.2 (29.0–37.1)*	32.4 (28.5–36.1)*	37.4 (30.8–42.0)*	35.6 (29.6–37.3)*	17.7 (16.9–17.7)*
MIR (BR)	4 [4–4]*	0 [0]	0 [0]	10.2 (9.5–11.6)*	11.5 (10.0–12.3)*	11.7 (10.0–13.6)*	13.0 (11.4–14.8)*	6.6 (5.7–7.3)*
MIR (ST)	4 [4–4]*	0 [0]	0 [0]	7.2 (6.2–8.2)*	7.5 (7.0–8.0)*	7.7 (6.0–9.5)*	9.1 (7.6–10.9)*	4.2 (4.0–7.3)*
40% reduced dose								
FBP	3 [3–3]	3 [16]	15 [79]	70.0 (62.8–83.5)*	72.2 (63.5–79.3)*	77.3 (69.6–97.0)*	74.8 (55.6–83.8)*	31.7 (25.9–37.7)*
iDose^4^	3 [3–4]	0 [0]	8 [42]	37.8 (34.6–39.7)*	38.0 (34.2–42.4)*	44.0 (37.7–45.7)*	39.4 (36.2–44.2)*	23.6 (20.0–28.6)
MIR (BR)	4 [3–4]*	0 [0]	4 [21]	12.4 (10.2–12.8)*	11.6 (9.8–14.4)*	13.6 (11.4–15.7)*	13.5 (11.2–17.6)*	7.5 (5.9–8.6)*
MIR (ST)	4 [3–4]*	0 [0]	2 [11]	8.4 (7.3–9.8)*	9.3 (7.3–10.7)*	10.2 (9.4–11.3)*	10.5 (8.9–14.3)*	5.6 (4.9–7.9)*
60% reduced dose								
FBP	2 [2–3]*	13 [68]	19 [100]	97.5 (84.1–106.7)*	88.7 (77.4–120.8)*	103.1 (89.8–115.0)*	103.7 (85.6–111.1)*	42.2 (33.0–49.8)*
iDose^4^	3 [3–3]	0 [0]	6 [32]	40.6 (38.9–45.1)*	39.3 (35.8–48.1)*	46.2 (40.2–52.5)*	45.6 (38.3–49.5)*	27.4 (24.5–37.2)*
MIR (BR)	4 [3–4]	1 [5]	7 [37]	13.7 (11.2–15.6)*	12.9 (10.8–14.6)*	15.2 (13.1–17.4)*	14.2 (12.5–16.4)*	8.5 (7.3–11.7)*
MIR level 2 (ST)	3 [3–4]*	0 [0]	2 [5]	10.1 (8.3–12.0)*	9.2 (8.6–10.7)*	11.6 (10.1–13.3)*	10.2 (9.2–12.3)*	7.2 (5.3–10.5)*
80% reduced dose								
FBP	1 [1–1]*	19 [100]	19 [100]	160.9 (136.0–187.6)*	168.7 (131.0–192.9)*	184.7 (152.8–203.3)*	165.6 (146.9–187.5)*	57.2 (36.8–63.2)*
iDose^4^	2 [2–3]*	11 [58]	17 [90]	47.4 (45.3–64.7)	51.0 (45.4–59.0)	50.6 (44.3–63.6)	51.8 (48.0–64.7)	33.4 (26.8–41.7)*
MIR (BR)	3 [3–3]*	4 [21]	14 [74]	15.9 (12.9–17.2)*	14.6 (12.3–17.8)*	17.1 (15.6–19.4)*	15.2 (13.9–19.1)*	9.8 (8.8–15.4)*
MIR (ST)	3 [2–3]*	6 [32]	13 [69]	12.4 (9.9–13.9)*	11.8 (10.3–14.7)*	14.4 (11.8–16.0)*	12.5 (10.5–15.7)*	9.2 (6.4–15.1)*

*Significant difference compared to the reference (p<0.0125)

*FBP* filtered back projection, *HIR* hybrid iterative reconstruction, *MIR* model-based iterative reconstruction*, BR* body routine, *ST* soft tissue, *Obs* observer

Overall agreement for subjective image quality was moderate with a weighted kappa of 0.59. The percentage of total agreement was 47%. In 4.3% of comparisons there was more than 1 point difference in the subjective image quality score between observers.

Noise and attenuation are presented in Table 3 and in the supplemental files in the Appendix (Table D, Fig. B-C). At routine dose, both HIR and MIR resulted in a reduction in noise. Reducing the radiation dose led to a significant increase in noise with FBP, while MIR resulted in a decrease in noise compared to FBP at routine dose, even at 80% reduced dose. With a 40% or 60% reduction in dose, HIR resulted in less noise compared to FBP at routine dose, while at 80% reduced dose the amount of noise was comparable to FBP at routine dose.

Mean densities were not affected by dose reduction, except for the lowest dose level and the density of air. This resulted in a slightly higher attenuation.

## Discussion

This prospective, within-patient study showed that the radiation dose can be reduced by 40% (to median 3.8 mGy) using HIR and by 60% (to median 2.6 mGy) using MIR in CT scans for urolithiasis evaluation in patients with a median weight of 86 kg and a BMI range from 20 to 39 kg/m^2^. Sensitivity for stones remained excellent at 60% reduced dose with IR while sensitivity decreased with low-dose FBP. At these low-dose levels, extra-urinary tract pathology was still assessable with IR, while objective and subjective image quality improved compared to FBP at routine dose. Further dose reduction hampered the diagnosis of extra-urinary tract pathology and is therefore not advised with current reconstruction methods.

Current guidelines recommend the use of low-dose CT in patients suspected of urolithiasis [4, 6]. The definition of low dose is not unambiguous, but a dose below 3 mSv is usually considered a low dose for native abdominal CT for follow-up of urolithiasis [14]. A large survey study performed in 93 hospitals in the USA between 2011 and 2013 including 49,903 renal colic CT examinations reported a mean radiation dose of 11.2 mSv (14.3 mGy) in clinical practice [15]. Only 2% of those examinations were performed at a low dose (<3 mSv) and 0.2% at a dose below 2 mSv. The high contrast between stones and the surrounding soft tissue should make it possible to substantially reduce the radiation dose without affecting diagnostic accuracy, and various studies have shown that the radiation dose for urinary stone CT acquisitions can be safely reduced below 3 mSv without affecting the diagnostic accuracy of stone detection [16]. One of the largest studies (201 patients) was performed by Moore and colleagues [8]. Patients suspected of ureteral stones received both a routine and low-dose CT acquisition with a radiation dose of 12.7 mSv and 1.6 mSv, respectively. The sensitivity at low dose was 90% with a specificity of 99%. There were 102 stones present, of which 75% were smaller than 5 mm. No IR was used. Fontarensky et al. [9] compared routine dose acquisitions with hybrid IR (ASIR, GE Healthcare) to low-dose acquisitions with model-based IR (MBIR, GE Healthcare) at a radiation dose level of 8.8 mSv (10.9 mGy) and 1.4 mSv (1.7 mGy), respectively. Both acquisitions were made successively in the same patients. The diagnostic accuracy at low dose was excellent and objective and subjective image quality were comparable. Also the detection of alternative diagnoses was not hampered at reduced dose. To our best knowledge, only two studies investigated ultra-low-dose acquisitions at submillisievert dose levels [10, 17]. In a study by Glazer et al. [10], a split-dose design was used in which 52 patients received both a 80% dose scan and a 20% dose scan. Radiation dose was 3.9 mSv (4.8 mGy) and 1.0 mSv (1.2 mGy), respectively, and MBIR was used for reconstruction. Subjective and objective image quality were significantly lower at reduced dose, and the diagnostic accuracy decreased to a sensitivity of 74% and a specificity of 77% for stones smaller than 3 mm. In a similar study by McLaughlin and colleagues [17], patients received a routine dose (4.4 mSv) and low-dose (0.5 mSv) acquisition. The sensitivity decreased to 72% at low dose, which was mainly caused by missed small stones. In addition, several extra-urinary findings like gallstones and appendicitis were missed at low dose. The current study corroborates those results and underscores that excessive radiation dose reduction to submillisievert dose levels is not feasible in an average adult due to a decrease in diagnostic accuracy. This study also found that the decrease in sensitivity is caused by small stones (<3 mm) that are missed, while larger stones remain visible.

One of the disadvantages of IR often mentioned is the longer reconstruction time compared to FBP [18]. This is mainly a problem of MIR algorithms, while HIR results in less than a minute delay compared to FBP [19]. The MIR algorithm used in the current study, IMR, takes less than 5 min for the majority of the protocols according to the vendor [20]. A more recent study by Yuki et al. investigating chest CT reported a reconstruction time within 3 min for all cases [21]. This delay is clinically acceptable for CT scans for urolithiasis. However, longer reconstruction times up to an hour have been reported for other MIR algorithms [22–24].

The main strength of the current study is the within-patient design using four different dose levels to investigate the achievable radiation dose reduction. Not only the accuracy for stone detection was researched, but also the possibility to diagnose extra-urinary tract pathology, which is important in clinical practice. Furthermore, both hybrid and more advanced model-based IR were investigated and, to our best knowledge, this is the first study using IMR for this purpose. This study has several limitations. First, a relatively small sample size was used because the study participants were exposed to four CT scans. However, compared to other studies a large number of stones was present. Second, the IR algorithms of only one vendor were investigated. Lastly, the effect of dose reduction and IR on the stone size was not investigated. A previous study showed that MIR might overestimate the stone density and size compared to HIR [25]. It was, however, not clear if this was truly an overestimation, or if HIR underestimated the stone size. Future studies using a phantom with stones with known density and size should be performed to demonstrate this.

In conclusion, the radiation dose for the assessment of urolithiasis can be reduced by 40% (HIR) to 60% (MIR) without affecting diagnostic performance or image quality. Further dose reduction leads to decreased sensitivity for small stones and hampers the assessment of extra-urinary tract pathology.

## Electronic supplementary material

Below is the link to the electronic supplementary material.

Table A Overall sensitivity for stone detection. The sensitivity was calculated per observer and stones, papillary calcifications and parenchymal calcifications were combined. *FBP* filtered back projection, *HIR* hybrid iterative reconstruction, *MIR* model-based iterative reconstruction, *BR* body routine, *ST* soft tissue, *Obs* observer (DOCX 14 kb)

Table B Aorta and adrenal gland measurements. The average value of both observers is used. There were no significant differences; however, several reconstructions were not assessable due to excessive noise.*FBP* filtered back projection, *HIR* hybrid iterative reconstruction, *MIR* model-based iterative reconstruction, *BR* body routine, *ST* soft tissue, *NA* not assessable (DOCX 19 kb)

Table C Agreement between observers for subjective measurements. *A weighted kappa was used for the subjective image quality score (DOCX 16 kb)

Table D Organ attenuation as a function of reconstruction method and dose level. Variables are presented as medians (interquartiles). *Significant difference compared to the reference (p<0.0125). *FBP* filtered back projection, *HIR* hybrid iterative reconstruction, *MIR* model-based iterative reconstruction, *BR* body routine, *ST* soft tissue, *NA* not assessable (DOCX 18 kb)

Fig. A Additional example of the subjective image quality score. From left to right the different scores: score 1 with FBP at 80% reduced dose, score 2 with MIR Soft Tissue at 80% reduced dose, score 3 with HIR at 60% reduced dose and score 4 with MIR Body Routine at the routine dose level (GIF 1221 kb)

High resolution image (TIF 14273 kb)

Fig. B Image noise as a function of dose level measured in different areas. Reference line represents the median noise with FBP at routine dose. *FBP* filtered back projection, *HIR* hybrid iterative reconstruction, *MIR* model-based iterative reconstruction (GIF 12 kb) (GIF 12 kb) (GIF 12 kb) (GIF 12 kb) (GIF 13 kb)

High resolution image (TIF 992 kb) High resolution image (TIF 992 kb) High resolution image (TIF 992 kb) High resolution image (TIF 992 kb) High resolution image (TIF 992 kb)

Fig. C Attenuation as a function of dose level measured in different areas. Reference line represents the median attenuation with FBP at routine dose. *FBP* filtered back projection, *HIR* hybrid iterative reconstruction, *MIR* model-based iterative reconstruction (GIF 15 kb) (GIF 15 kb) (GIF 16 kb) (GIF 15 kb) (GIF 16 kb)

High resolution image (TIF 992 kb) High resolution image (TIF 992 kb) High resolution image (TIF 992 kb) High resolution image (TIF 992 kb) High resolution image (TIF 992 kb)

## Abbreviations

CT: Computed tomography
CTDIvol: Volumetric CT dose index
DLP: Dose length product
FBP: Filtered back projection
HIR: Hybrid iterative reconstruction
HU: Hounsfield unit
ICC: Intraclass correlation coefficient
IQR: Interquartile range
IR: Iterative reconstruction
MIR: Model-based iterative reconstruction
ROI: Region of interest
SD: Standard deviation
