# Supplementary material for: Radiation dose reduction for CT assessment of urolithiasis using iterative reconstruction: A prospective intra-individual study
Source: Eur Radiol. 2017 Jul 10;28(1):143–50. doi: 10.1007/s00330-017-4929-2 (PMC5717126; doi:10.1007/s00330-017-4929-2)
Supplement: Supplementary file 1 — Overall sensitivity for stone detection. The sensitivity was calculated per observer and stones, papillary calcifications and parenchymal calcifications were combined. FBP filtered back projection, HIR hybrid iterative reconstruction, MIR model-based iterative reconstruction, BR body routine, ST soft tissue, Obs observer (DOCX 14 kb) [file 330_2017_4929_MOESM1_ESM.docx]

**Table A –** Overall sensitivity for stone detection and the number of false positives. The sensitivity was calculated per observer and stones, papillary calcifications and parenchymal calcifications were combined. *FBP Filtered Back Projection, HIR Hybrid Iterative Reconstruction, MIR Model-based Iterative Reconstruction, BR Body Routine, ST Soft Tissue, Obs Observer*

|  | Sensitivity | | False positives | |
| --- | --- | --- | --- | --- |
|  | Obs. 1 | Obs. 2 | Obs. 1 | Obs. 2 |
| Routine dose |  |  |  |  |
| FBP | 89% | 86% | 18 | 8 |
| HIR | 88% | 86% | 10 | 26 |
| MIR BR | 95% | 95% | 25 | 32 |
| MIR ST | 92% | 89% | 23 | 23 |
| 40% reduced dose |  |  |  |  |
| FBP | 86% | 76% | 6 | 15 |
| HIR | 91% | 85% | 6 | 23 |
| MIR BR | 91% | 92% | 17 | 40 |
| MIR ST | 91% | 93% | 15 | 15 |
| 60% reduced dose |  |  |  |  |
| FBP | 76% | 64% | 19 | 6 |
| HIR | 83% | 80% | 11 | 12 |
| MIR BR | 88% | 88% | 19 | 27 |
| MIR ST | 89% | 88% | 6 | 19 |
| 80% reduced dose |  |  |  |  |
| FBP | 42% | 46% | 5 | 5 |
| HIR | 70% | 73% | 5 | 9 |
| MIR BR | 78% | 76% | 5 | 15 |
| MIR ST | 73% | 81% | 4 | 7 |
