# Supplementary material for: Radiation dose reduction for CT assessment of urolithiasis using iterative reconstruction: A prospective intra-individual study
Source: Eur Radiol. 2017 Jul 10;28(1):143–50. doi: 10.1007/s00330-017-4929-2 (PMC5717126; doi:10.1007/s00330-017-4929-2)
Supplement: Supplementary file 2 — Aorta and adrenal gland measurements. The average value of both observers is used. There were no significant differences; however, several reconstructions were not assessable due to excessive noise.FBP filtered back projection, HIR hybrid iterative reconstruction, MIR model-based iterative reconstruction, BR body routine, ST soft tissue, NA not assessable (DOCX 19 kb) [file 330_2017_4929_MOESM2_ESM.docx]

**Table B –** Aorta and adrenal gland measurements. The average value of both observers is used. There were no significant differences, however several reconstructions were not assessable due to excessive noise. *FBP Filtered Back Projection, HIR Hybrid Iterative Reconstruction, MIR Model-based Iterative Reconstruction, BR Body Routine, ST Soft Tissue, NA Not Assessable*

|  | Aorta diameter | Left adrenal gland – size (mm) | Left adrenal gland – density (HU) | Right adrenal gland – size (mm) | Right adrenal gland – density (HU) |
| --- | --- | --- | --- | --- | --- |
| Routine dose |  |  |  |  |  |
| FBP | 22.0 [20.7 – 25.0] | 6.9 [5.7 – 8.1] | 17.0 [6.5 – 26.8] | 5.9 [5.3 – 6.8] | 25.8 [8.0 – 30.7] |
| HIR | 21.3 [20.1 – 24.7] | 7.1 [4.8 – 7.8] | 28.7 [11.9 – 37.2] | 6.2 [4.9 – 6.9]  1 NA | 27.8 [17.6 – 33.4]  1 NA |
| MIR (BR) | 21.6 [20.0 – 25.0] | 6.5 [5.4 – 8.3] | 25.1 [21.3 – 30,1] | 5.5 [5.0 – 6.3] | 25.8 [16.6 – 37.5] |
| MIR (ST) | 21.1 [19.6 – 25.8] | 6.1 [5.4 – 7.4] | 22.2 [16.3 – 31.0] | 5.8 [5.3 – 6.7] | 24.3 [15.8 – 32.4] |
| 40% reduced dose |  |  |  |  |  |
| FBP | 21.5 [19.7 – 25.1] | 6.2 [5.6 – 6.9]  3 NA | 23.8 [15.9 – 33.9]  3 NA | 5.5 [5.2-5.9]  3 NA | 29.8 [16.4 – 47.6]  3 NA |
| HIR | 22.0 [20.2 – 25.7] | 6.8 [5.8 – 7.1] | 20.6 [14.8 – 26.1] | 5.6 [5.4-6.4] | 29.9 [12.0 – 34.3] |
| MIR (BR) | 21.1 [20.2 – 25.6] | 5.8 [5.1 – 7.6] | 27.3 [18.2 – 34.7] | 5.6 [4.8-6.6] | 22.2 [20.4 – 30.8] |
| MIR (ST) | 21.4 [20.3 – 25.4] | 6.7 [5.5 – 7.0] | 18.9 [14.3 – 29.7] | 5.9 [5.2-6.5] | 21.1 [10.0 – 25.2] |
| 60% reduced dose |  |  |  |  |  |
| FBP | 21.6 [19.7 – 25.1] | 7.2 [6.7 – 7.9]  13 NA | 23.5 [17.6 -41.9]  13 NA | 6.3 [6.2 – 6.8]  14 NA | 27.5 [25.0 – 38.1]  14 NA |
| HIR | 21.7 [20.1 – 25.2] | 6.6 [6.0 – 7.7]  1 NA | 21.1 [9.8 – 37.2]  1 NA | 5.7 [5.3 – 6.2]  1 NA | 23.2 [14.2 – 37.9]  1 NA |
| MIR (BR) | 21.2 [20.5 – 25.0] | 6.6 [5.7 – 7.4] | 24.2 [11.8 – 32.2] | 5.7 [5.2 – 6.8] | 25.4 [17.5 – 34.8] |
| MIR (ST) | 21.4 [20.4 – 24.5] | 6.4 [5.7 – 6.9] | 19.9 [14.0 – 32.8] | 5.6 [5.2 – 6.3] | 25.4 [13.0 – 31.2] |
| 80% reduced dose |  |  |  |  |  |
| FBP | 21.3 [19.8 – 24.8]  2 NA | 19 NA | 19 NA | 19 NA | 19 NA |
| HIR | 22.6 [20.3 – 24.9] | 7.0 [6.1 – 7.8]  7 NA | 29.1 [18.2 – 52.6]  7 NA | 5.7 [5.5 – 6.4]  10 NA | 46.5 [23.7 – 48.2]  10 NA |
| MIR (BR) | 22.0 [20.5 – 24.3]  1 NA | 6.2 [5.7 – 7.1]  1 NA | 20.4 [8.7 – 30.6]  1 NA | 5.6 [5.1 – 6.3]  3 NA | 30.7 [22.9 – 42.7]  3 NA |
| MIR (ST) | 22.0 [19.7 – 24.7] | 6.3 [5.7 – 6.9]  1 NA | 22.1 [15.6 – 32.3]  1 NA | 5.8 [4.8 – 6.3]  3 NA | 27.2 [14.9 – 37.6]  3 NA |
