# Supplementary material for: Radiation dose reduction for CT assessment of urolithiasis using iterative reconstruction: A prospective intra-individual study
Source: Eur Radiol. 2017 Jul 10;28(1):143–50. doi: 10.1007/s00330-017-4929-2 (PMC5717126; doi:10.1007/s00330-017-4929-2)
Supplement: Supplementary file 3 — Agreement between observers for subjective measurements. *A weighted kappa was used for the subjective image quality score (DOCX 16 kb) [file 330_2017_4929_MOESM3_ESM.docx]

**Table C -** Agreement between observers for subjective measurements. **A weighted kappa was used for the subjective image quality score*

|  | Kappa | Total agreement (%) |
| --- | --- | --- |
| Subjective image quality | 0.59* | 46.7 |
| Prior cholecystectomy | 0.91 | 99.0 |
| Gall bladder stones | 0.87 | 94.4 |
| Gall bladder wall thickening | 0.31 | 64.1 |
| Sigmoid diverticulitis | 0.53 | 95.7 |
| Appendix visible | 0.66 | 79.9 |
| Appendicitis | 0.44 | 85.9 |
|  |  |  |
|  | **ICC** | **Difference** |
| Aorta diameter | 0.95 | -0.42 |
| Left adrenal gland – size (mm) | 0.18 | -1.23 |
| Left adrenal gland – density (HU) | 0.00 | 3.91 |
| Right adrenal gland – size (mm) | 0.27 | -0.18 |
| Right adrenal gland – density (HU) | 0.39 | 7.53 |
