# Supplementary material for: Radiation dose reduction for CT assessment of urolithiasis using iterative reconstruction: A prospective intra-individual study
Source: Eur Radiol. 2017 Jul 10;28(1):143–50. doi: 10.1007/s00330-017-4929-2 (PMC5717126; doi:10.1007/s00330-017-4929-2)
Supplement: Supplementary file 4 — Organ attenuation as a function of reconstruction method and dose level. Variables are presented as medians (interquartiles). *Significant difference compared to the reference (p<0.0125). FBP filtered back projection, HIR hybrid iterative reconstruction, MIR model-based iterative reconstruction, BR body routine, ST soft tissue, NA not assessable (DOCX 18 kb) [file 330_2017_4929_MOESM4_ESM.docx]

|  | Renal cortex (right) | Renal cortex (left) | Aorta | Retroperitoneal fat | Air |
| --- | --- | --- | --- | --- | --- |
| Routine dose |  |  |  |  |  |
| FBP | 36.3 (33.0 – 40.3)  *Reference* | 35.7 (32.1 – 39.0)  *Reference* | 50.7 (48.4 – 55.3)  *Reference* | -105.0 (-109.9 – -99.3)  *Reference* | -996.3 (-998.1 – -994.4)  *Reference* |
| HIR | 35.9 (33.5 – 39.6) | 36.3 (33.9 – 38.3) | 50.1 (47.9 – 54.6) | -105.4 (-109.5 – -99.8) | -997.5 (-1000.9 – -992.5) |
| MIR (BR) | 36.2 (33.3 – 39.3) | 36.0 (33.0 – 37.1) | 53.1 (46.0 – 56.6) | -103.3 (-107.9 – -95.7) | -991.6 (-993.9 – -990.5)* |
| MIR (ST) | 37.6 (34.7 – 40.4) | 37.1 (34.7 – 38.9) | 54.1 (48.0 – 56.4) | -103.2 (-107.0 – -97.1) | -995.9 (-997.3 – -993.0) |
| 40% reduced dose |  |  |  |  |  |
| FBP | 37.3 (33.7 – 41.1) | 36.3 (33.4 – 39.9) | 52.5 (48.7 – 55.7) | -105.8 (-112.8 – -100.7) | -991.9 (-994.5 – -989.3) |
| HIR | 36.1 (31.2 – 39.7) | 33.6 (32.4 – 37.8) | 50.8 (47.6 – 55.1) | -106.3 (-110.6 – -99.0) | -993.6 (-996.8 – -992.3) |
| MIR (BR) | 36.8 (32.3 – 40.3) | 35.4 (33.3 – 39.3) | 53.2 (49.8 – 56.3) | -105.9 (-108.7 – -97.6) | -987.7 (-994.0 – -983.1)* |
| MIR (ST) | 37.3 (33.6 – 40.9) | 36.9 (34.6 – 40.6) | 54.2 (50.4 – 56.8) | -105.4 (-108.2 – -96.8) | -991.0 (-994.0 – -987.5)* |
| 60% reduced dose |  |  |  |  |  |
| FBP | 40.7 (34.4 – 48.6)* | 39.4 (35.9 – 42.1) | 52.9 (43.5 – 56.8) | -104.5 (-108.4 – -96.0) | -998.4 (-993.4 – -982.3)* |
| HIR | 36.5 (29.5 – 39.8) | 34.5 (30.3 – 39.9) | 49.6 (42.9 – 51.9) | -107.0 (-110.2 – -95.0) | -994.3 (-996.1 – -981.7) |
| MIR (BR) | 37.8 (32.5 – 38.9) | 37.8 (34.7 – 40.9) | 49.6 (45.5 – 55.8) | -105.7 (-109.7 – -96.8) | -983.0 (-988.4 – -977.3)* |
| MIR (ST) | 39.2 (33.7 – 40.1) | 39.2 (35.8 – 41.7) | 50.5 (46.2 – 56.6) | -105.4 (-108.9 – -95.7) | -988.1 (-993.2 – -984.2)* |
| 80% reduced dose |  |  |  |  |  |
| FBP | 49.2 (40.1 – 57.0)* | 46.9 (42.0 – 54.1)* | 63.6 (54.1 – 78.5)* | -101.4 (-109.7 – -89.6) | -984.5 (-996.3 – -979.6)* |
| HIR | 39.6 (32.6 – 45.2) | 36.7 (33.7 – 44.0) | 47.0 (40.6 – 55.5) | -104.6 (-110.8 – -95.3) | -992.1 (-996.4 – -985.7) |
| MIR (BR) | 39.9 (32.7 – 42.3) | 38.0 (34.2 – 41.6) | 52.9 (47.1 – 58.3) | -102.6 (-105.6 – -97.8) | -984.9 (-990.1 – -979.0)* |
| MIR (ST) | 41.1 (34.4 – 43.2)* | 39.0 (35.4 – 42.9) | 54.9 (48.7 – 59.1) | -102.0 (-104.4 – -97.0) | -988.1 (-994.4 – -982.0)* |

**Table D –** Organ attenuation as a function of reconstruction method and dose level. Variables are presented as medians (interquartiles). *Significant difference compared to the reference (p<0.0125)

*FBP filtered back projection, HIR Hybrid Iterative Reconstruction, MIR Model-based Iterative Reconstruction, BR Body Routine, ST Soft Tissue*
