# Supplementary figures and images for: Radiation dose reduction for CT assessment of urolithiasis using iterative reconstruction: A prospective intra-individual study
Source: Eur Radiol. 2017 Jul 10;28(1):143–50. doi: 10.1007/s00330-017-4929-2 (PMC5717126; doi:10.1007/s00330-017-4929-2)

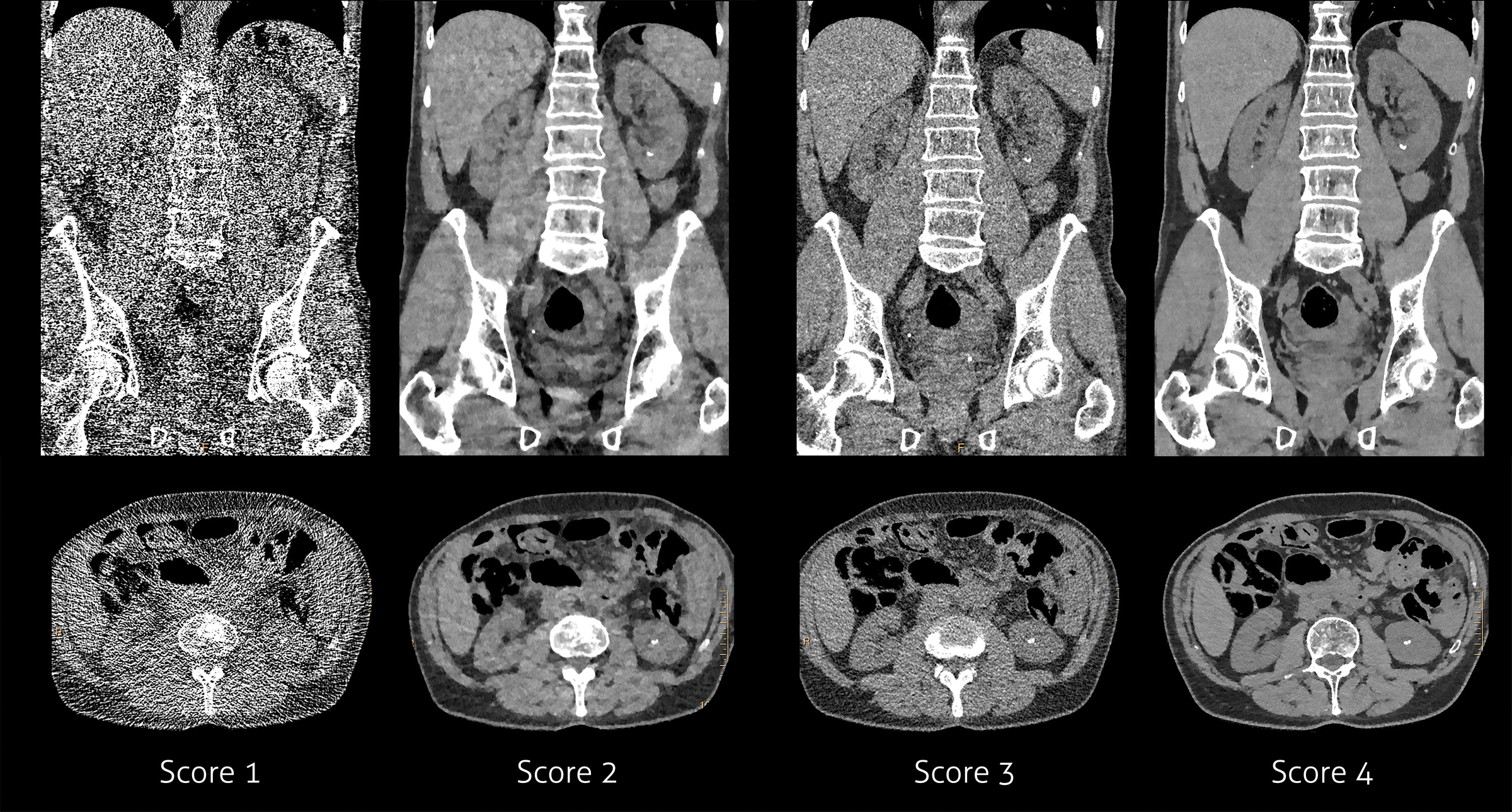

Supplement: Supplementary file 5 — Additional example of the subjective image quality score. From left to right the different scores: score 1 with FBP at 80% reduced dose, score 2 with MIR Soft Tissue at 80% reduced dose, score 3 with HIR at 60% reduced dose and score 4 with MIR Body Routine at the routine dose level (GIF 1221 kb) [file 330_2017_4929_Fig3_ESM.gif]

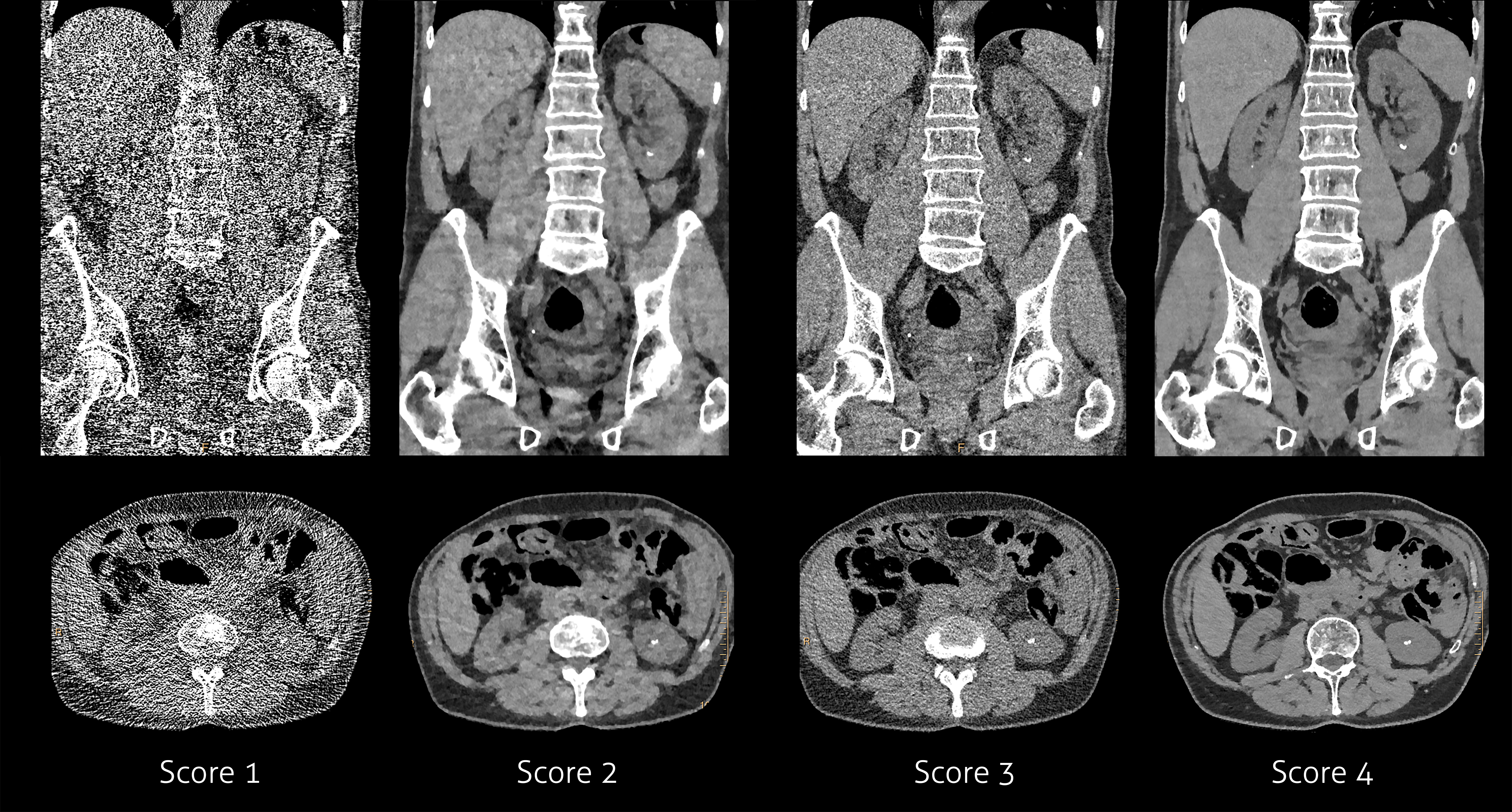

Supplement: Supplementary file 6 — High resolution image (TIF 14273 kb) [file 330_2017_4929_MOESM5_ESM.tif]

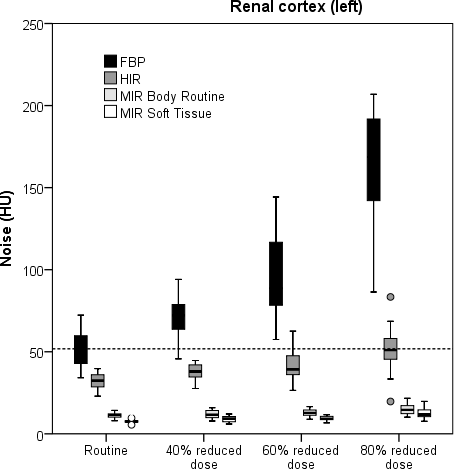

Supplement: Fig. B — Image noise as a function of dose level measured in different areas. Reference line represents the median noise with FBP at routine dose. FBP filtered back projection, HIR hybrid iterative reconstruction, MIR model-based iterative reconstruction (GIF 12 kb) [file 330_2017_4929_Fig4_ESM.gif]

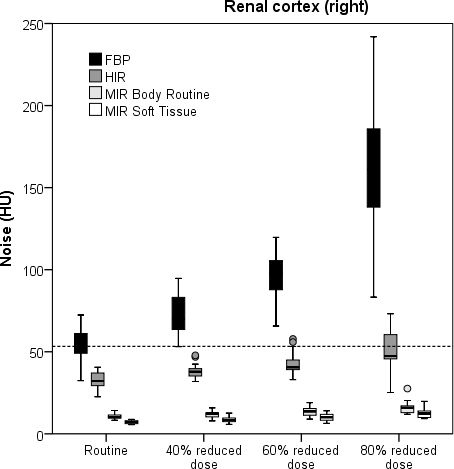

Supplement: Fig. B — Image noise as a function of dose level measured in different areas. Reference line represents the median noise with FBP at routine dose. FBP filtered back projection, HIR hybrid iterative reconstruction, MIR model-based iterative reconstruction (GIF 12 kb) [file 330_2017_4929_Fig5_ESM.gif]

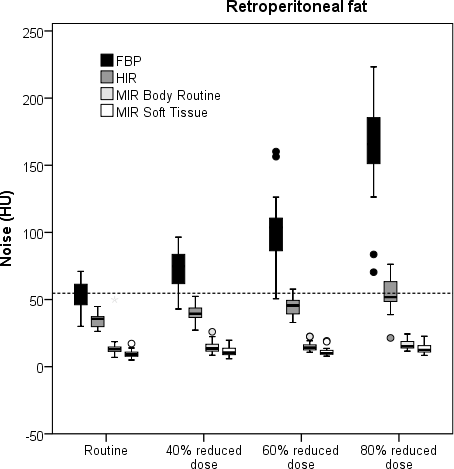

Supplement: Fig. B — Image noise as a function of dose level measured in different areas. Reference line represents the median noise with FBP at routine dose. FBP filtered back projection, HIR hybrid iterative reconstruction, MIR model-based iterative reconstruction (GIF 12 kb) [file 330_2017_4929_Fig6_ESM.gif]

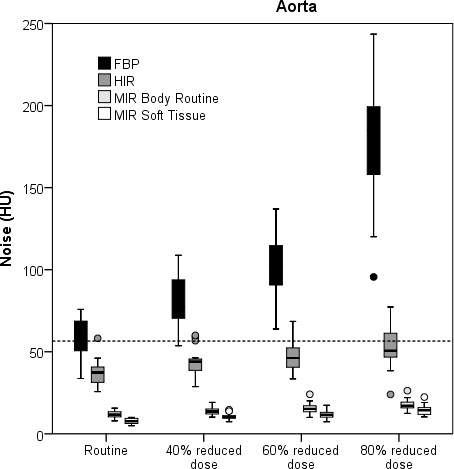

Supplement: Fig. B — Image noise as a function of dose level measured in different areas. Reference line represents the median noise with FBP at routine dose. FBP filtered back projection, HIR hybrid iterative reconstruction, MIR model-based iterative reconstruction (GIF 12 kb) [file 330_2017_4929_Fig7_ESM.gif]

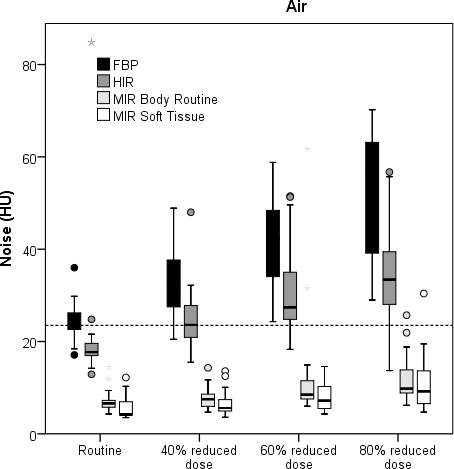

Supplement: Fig. B — Image noise as a function of dose level measured in different areas. Reference line represents the median noise with FBP at routine dose. FBP filtered back projection, HIR hybrid iterative reconstruction, MIR model-based iterative reconstruction (GIF 12 kb) [file 330_2017_4929_Fig8_ESM.gif]

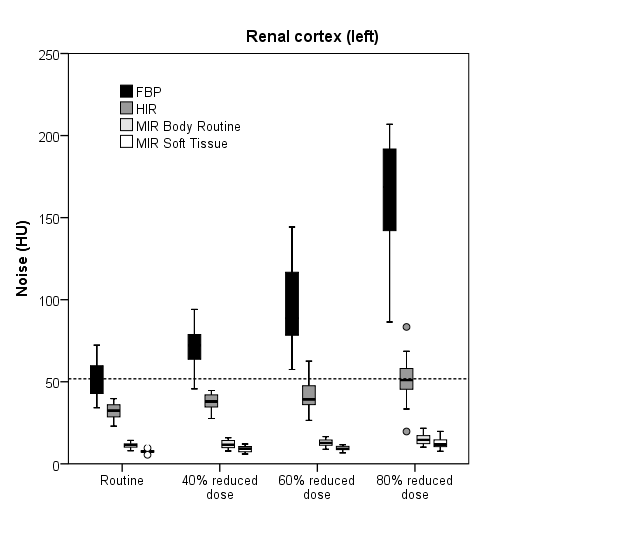

Supplement: Supplementary file 12 — High resolution image (TIF 992 kb) [file 330_2017_4929_MOESM6_ESM.tif]

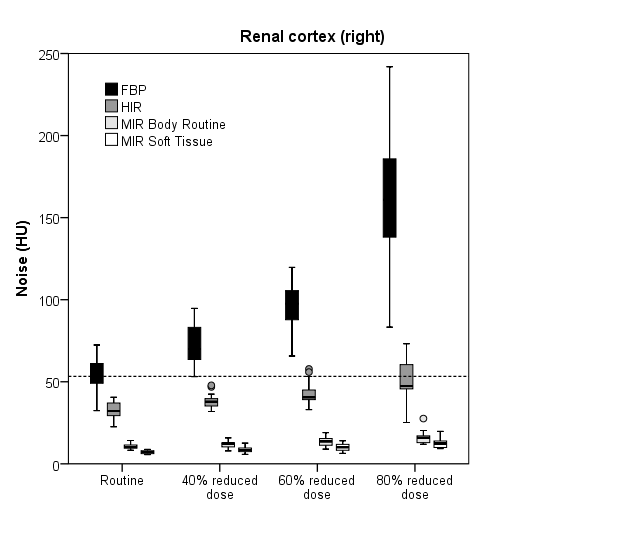

Supplement: Supplementary file 13 — High resolution image (TIF 992 kb) [file 330_2017_4929_MOESM7_ESM.tif]

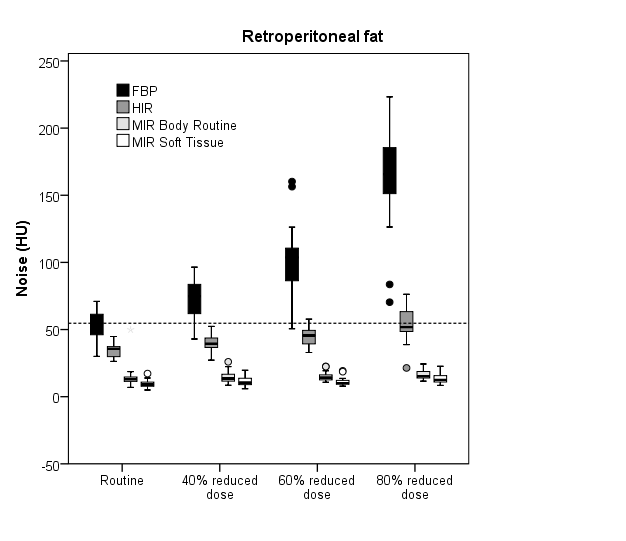

Supplement: Supplementary file 14 — High resolution image (TIF 992 kb) [file 330_2017_4929_MOESM8_ESM.tif]

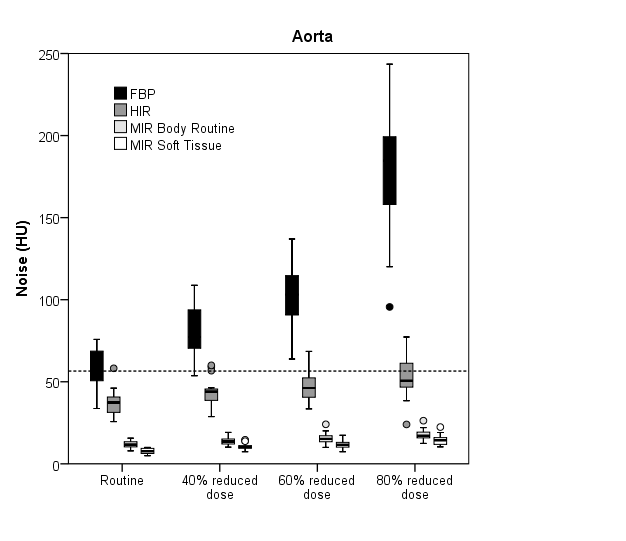

Supplement: Supplementary file 15 — High resolution image (TIF 992 kb) [file 330_2017_4929_MOESM9_ESM.tif]

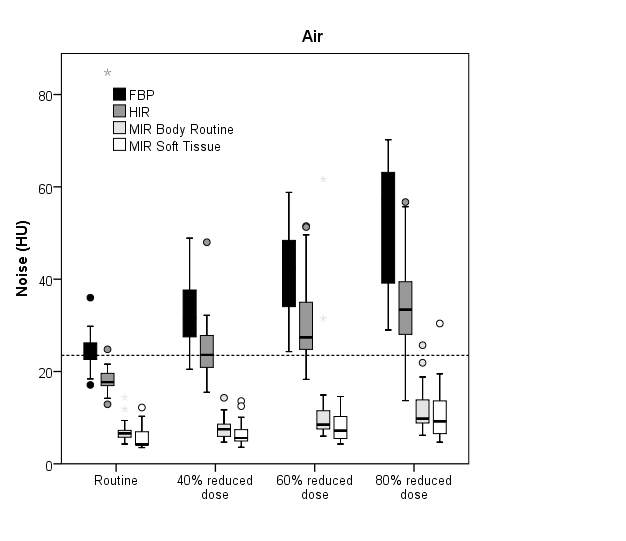

Supplement: Supplementary file 16 — High resolution image (TIF 992 kb) [file 330_2017_4929_MOESM10_ESM.tif]

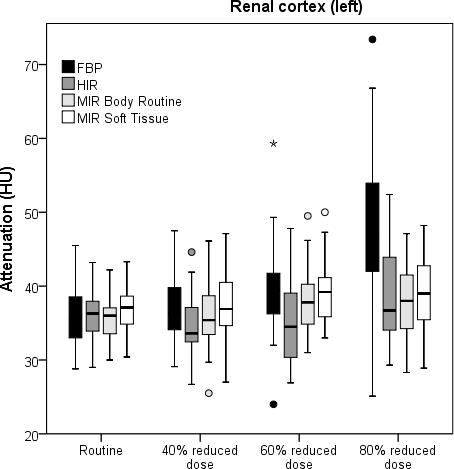

Supplement: Fig. C — Attenuation as a function of dose level measured in different areas. Reference line represents the median attenuation with FBP at routine dose. FBP filtered back projection, HIR hybrid iterative reconstruction, MIR model-based iterative reconstruction (GIF 15 kb) [file 330_2017_4929_Fig9_ESM.gif]

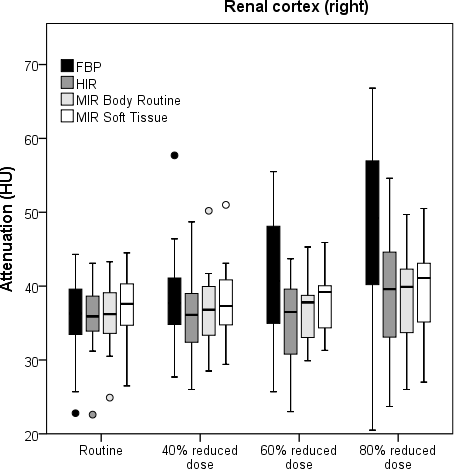

Supplement: Fig. C — Attenuation as a function of dose level measured in different areas. Reference line represents the median attenuation with FBP at routine dose. FBP filtered back projection, HIR hybrid iterative reconstruction, MIR model-based iterative reconstruction (GIF 15 kb) [file 330_2017_4929_Fig10_ESM.gif]

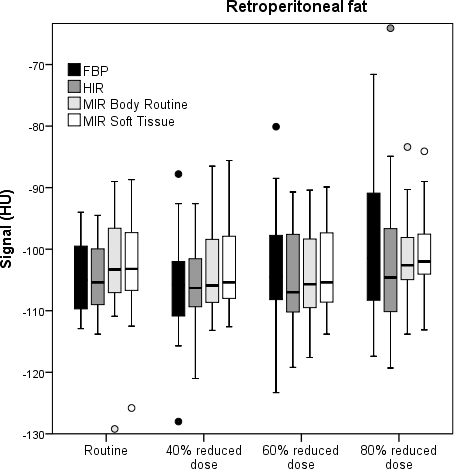

Supplement: Fig. C — Attenuation as a function of dose level measured in different areas. Reference line represents the median attenuation with FBP at routine dose. FBP filtered back projection, HIR hybrid iterative reconstruction, MIR model-based iterative reconstruction (GIF 15 kb) [file 330_2017_4929_Fig11_ESM.gif]

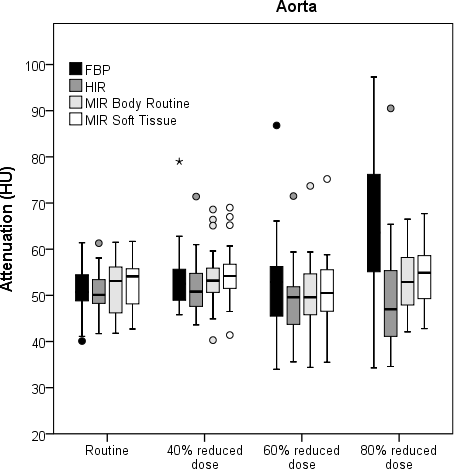

Supplement: Fig. C — Attenuation as a function of dose level measured in different areas. Reference line represents the median attenuation with FBP at routine dose. FBP filtered back projection, HIR hybrid iterative reconstruction, MIR model-based iterative reconstruction (GIF 15 kb) [file 330_2017_4929_Fig12_ESM.gif]

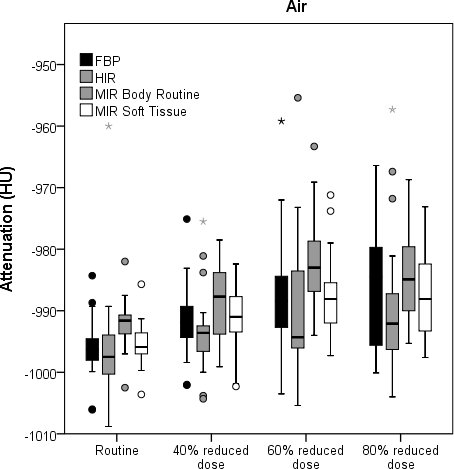

Supplement: Fig. C — Attenuation as a function of dose level measured in different areas. Reference line represents the median attenuation with FBP at routine dose. FBP filtered back projection, HIR hybrid iterative reconstruction, MIR model-based iterative reconstruction (GIF 15 kb) [file 330_2017_4929_Fig13_ESM.gif]

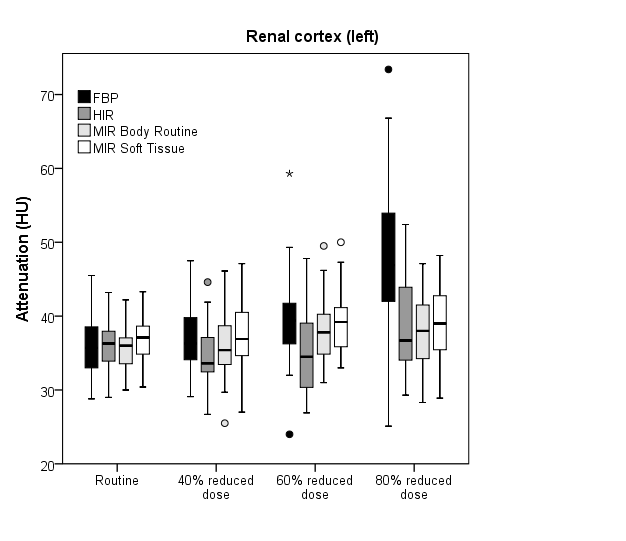

Supplement: Supplementary file 22 — High resolution image (TIF 992 kb) [file 330_2017_4929_MOESM11_ESM.tif]

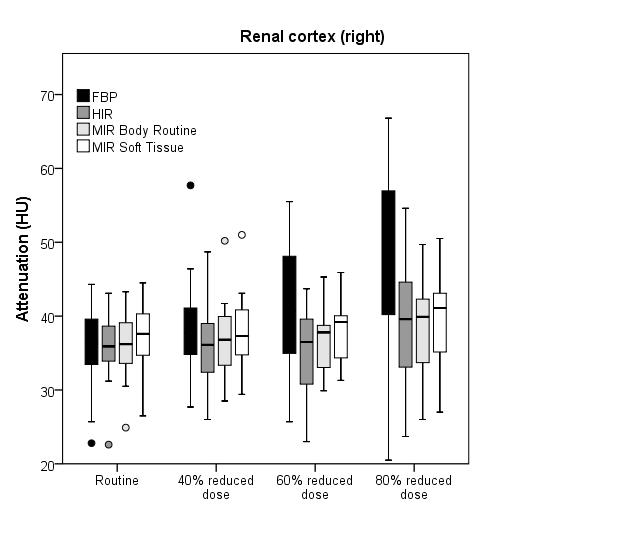

Supplement: Supplementary file 23 — High resolution image (TIF 992 kb) [file 330_2017_4929_MOESM12_ESM.tif]

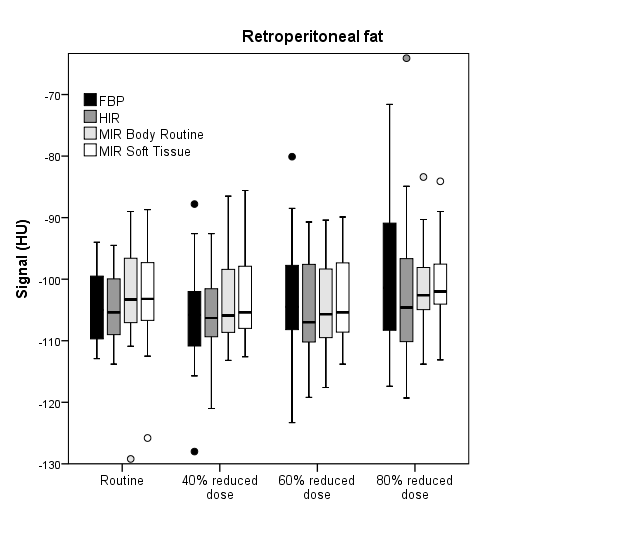

Supplement: Supplementary file 24 — High resolution image (TIF 992 kb) [file 330_2017_4929_MOESM13_ESM.tif]

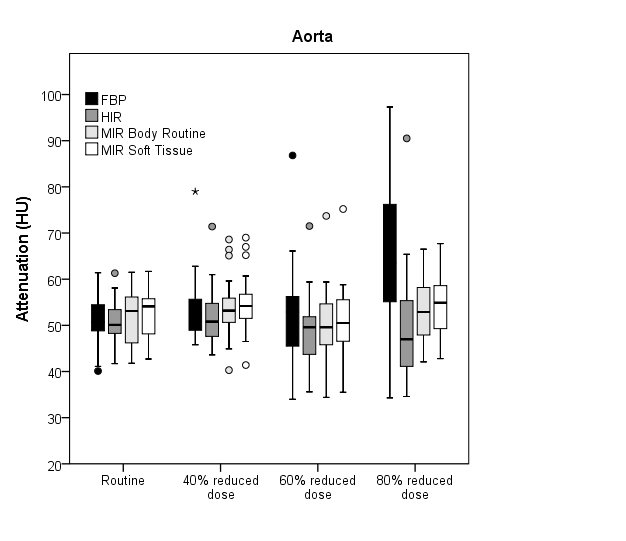

Supplement: Supplementary file 25 — High resolution image (TIF 992 kb) [file 330_2017_4929_MOESM14_ESM.tif]

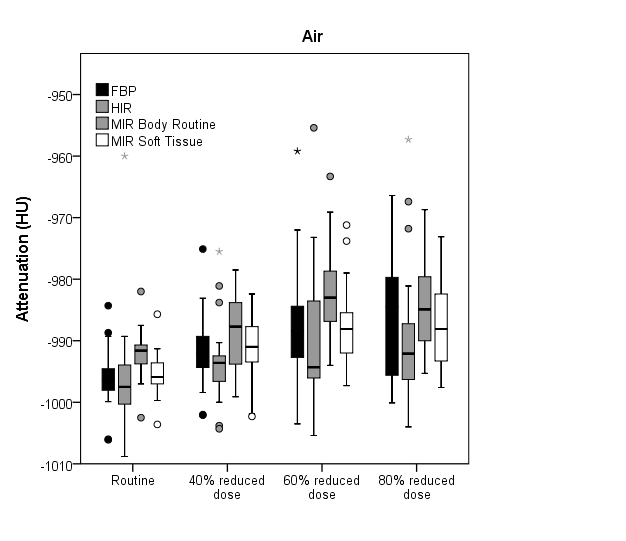

Supplement: Supplementary file 26 — High resolution image (TIF 992 kb) [file 330_2017_4929_MOESM15_ESM.tif]
